# Clinico-epidemiological evaluation of pharmaceutical/non-pharmaceutical poisoning in a referral poisoning emergency in the Central part of Iran

**DOI:** 10.1038/s41598-024-61411-w

**Published:** 2024-05-07

**Authors:** Nastaran Eizadi-Mood, Hamed Sanjari, Awat Feizi, Razieh Yazdi, Amin Dorostkar, Razieh Mahvari, Parisa Mirmoghtadaee, Rokhsareh Meamar

**Affiliations:** 1 Department of Clinical Toxicology, Khorshid hospital, School of Medicine, Isfahan Clinical Toxicology Research Center, Isfahan University of Medical Sciences, Isfahan, Iran; 2 Isfahan Clinical Toxicology Research Center, Isfahan University of Medical Sciences, Isfahan, Iran; 3 Department of Epidemiology & Biostatistics, School of Public Health, Isfahan University of Medical Sciences, Isfahan, Iran; 4 Department of Clinical Toxicology, Isfahan University of Medical Sciences, Isfahan, Iran; 5 Deputy of Research and Technology, Isfahan University of Medical Sciences, Isfahan, Iran; 6 Isfahan Clinical Toxicology Research Center, Khorshid hospital, Isfahan University of Medical Sciences, Ostandari Street, Hasht Behest Avenue, Isfahan, 81458-31451 Iran

**Keywords:** Poisoning, Pharmaceutical, Pharmaceutical substances, Medication, Medical research, Risk factors

## Abstract

The pattern of poisoning varies in different societies. In this study, we investigated the clinical-epidemiological features and outcomes of poisoned patients based on the substances involved, whether pharmaceutical or non- pharmaceutical toxins. This cross-sectional study involved a retrospective chart review of all poisoned patients who presented to the poisoning emergency hospital in the center of Iran between January 2015 and December 2019. We collected data on socio-demographics, the nature of the poisoning, and the outcomes. Backward stepwise binary regression analysis was conducted to predict the mortality. Throughout the study period, 5777 patients with acute poisoning met the inclusion criteria. Of these, 3524 cases (61%) were attributed to pharmaceutical, and 2253 cases (39%) were due to non-pharmaceutical poisoning. The majority of pharmaceutical poisonings (82.9%) were intentional, whereas non-pharmaceutical poisonings accounted for 46.2% of intentional exposures (P < 0.001). Patients with non-pharmaceutical poisoning were predominantly men, older in age, and had a history of addiction compared to those with pharmaceutical poisoning (P < 0.001). In binary logistic regression analysis, patients poisoned by non-pharmaceutical substances had a significantly higher risk of mortality [Odds ratio, 3.14; (95% CI 1.39–7.10); P = 0.006] compared to those poisoned by pharmaceutical substances (P < 0.001). The pattern of poisoning differs in terms of age and gender when comparing pharmaceutical to non-pharmaceutical poisoning. Patients poisoned by non-pharmaceutical may have a worse outcome compared to those poisoned by pharmaceutical substances.

## Introduction

Poisoning is a significant global health issue, with at least 500,000 people dying annually worldwide^1^. Because of its frequency, severity, potential for death and disability, and resulting hospitalization costs, it remains a major public health concern. The World Health Organization (WHO) estimates that eighty-four percent of deaths from unintentional poisoning occur in countries with low- and middle-income^2^. In 2019, the National Crime Records Bureau (NCRB) reported that about 5.1% of accidental deaths in India and 25.8% of suicides were related to poisoning^3^. In Iran, poisoning is a common cause of emergency admissions, has been estimated to be the second cause of death^3^ accounting for 2.5–5% of all deaths^4^. Also, the mortality rate from poisoning has been reported to be 8 per 1000 individuals in the general hospitalization wards and 109 per 1000 people in the intensive care unit (ICU) in another study in Iran^4^.

Poisoning can occur from pharmaceutical products, as well as non-pharmaceutical substances such as pesticides and household chemicals, which are common in many parts of the world^5–9^ In developed countries the most common cause of acute poisoning is the abuse of commercially available pharmaceuticals^10^. In contrast, in developing countries, insecticides are the most common cause^11^. A review study showed that poisoning with pharmaceutical compounds was common in most parts of Iran^7^. Also, in a survey conducted in the emergency department of Turkey, medication was the most prevalent substance^9^, while toxicities with alcohol and its derivatives have been reported in other studies^6,10,12^. Hadeiy et al. in a study in Iran from 2012 to 2018 reported that antiepileptic, sedative, and antiparkinson medicines were the most common substances, followed by narcotics and psychotropic drugs^13^. Non-pharmaceutical poisonings including organophosphorus compounds, aluminum phosphide, opium, and detergents are also common in developing countries^14,15^. In eastern Iran, a unique substance called Majoon Birjandi, which contains cannabis, is used and can lead to poisoning. Pesticide-related toxicities were more common in northern agricultural regions and specifically organophosphates and aluminum phosphide, remains a common hazard in Iran^14,15^. Pesticides are easily available to the general public in almost every country in the Asia Pacific region^16^.

Analyzing poisonings in a specific area is essential for identifying risk factors^9^. Comparing pharmaceutical and non-pharmaceutical poisoning is critical due to their unique challenges and implications. Pharmaceutical poisonings frequently involve intentional overdoses or medication errors with readily available substances^17^. Conversely, non-pharmaceutical poisonings often stem from occupational exposure or accidental ingestion of chemicals found in household products or pesticides^18^. Each poisoning type carries distinct implications for treatment, patient outcomes, and targeted preventative measures.

Given the differences in access to pharmaceutical and non-pharmaceutical substances among various communities, and the lack of comparative studies on this topic in Iran, we evaluated the clinical-epidemiological features and outcomes of poisoned patients involving both types of substances at a referral poisoning center. Our findings will provide a deeper understanding of the common agents, patient demographics, and outcomes in a healthcare setting, helping to identify specific needs for interventions and reduce hospital morbidity and mortality. This data is crucial for developing targeted prevention strategies and optimizing resource utilization, with the ultimate goal of reducing the economic burden of poisoning in our community. Furthermore, to determine significant differences in poisoning patterns among different age groups, we conducted a comparative analysis.

## Materials and methods

### Setting and patients

The current study was a hospital-based cross-sectional study that consisted of a retrospective chart review. In order to ensure the highest volume of data for analysis, we utilized a retrospective chart in a cross-sectional study. It included all poisoned patients who were admitted to the referral poisoning emergency center of Khorshid Hospital, which is affiliated with Isfahan University of Medical Sciences in Isfahan, Iran, from January 2015 to December 2019. Any patient who had exposure to pharmaceutical or non-pharmaceutical substances and visited by the emergency department staff in the poisoning emergency department even the patient was symptom free was included in the study. Exclusion criteria included and the discharge of patients with personal consent (patients who, after recovering from poisoning, did not wait for psychiatric evaluation and chose to be discharged). The study was conducted in accordance with the Declaration of Helsinki and was approved by the ethics committee of Isfahan University of Medical Sciences (Ethics code: IR.MUI.MED.REC.1397.316). An informed consent form was obtained from all participants. We considered the following criteria for informed consent (1) disclosure of information, (2) competency of the patient (or surrogate) and (3) the consent was voluntary. And to keep their patient’s identity confidentially, we used password protected files to keep their records secure.

### Data collection

The data collection was based on the ICD‑10 code^19^*.* The T36–T50 (drugs, medicaments, and biological substances) and T51–T65 (toxic effects of substances chiefly non-medicinal) of ICD-10 codes were used for the classification of patient diagnosis. The attending physicians made the diagnosis of poisoning based on the history reported by the patients or their relatives, clinical manifestations, serum/blood toxicological tests, and toxicology urine analysis, if necessary. The sources of data were medical records, laboratory results, and interviews. To ensure the quality of data, we checked the availability of variables on some randomly selected medical charts to ensure the agreement of the data abstraction format that satisfied the objective of our study. Then amendments were conducted. Data collectors and supervisors were recruited based on their experience in research. Principal investigator trained the objective of the study, data collection tool, and data collection procedures Supervision was conducted by the principal investigators who also reviewed the collected data and checked for completeness, accuracy, and consistency immediately after collection and appropriately arranged and kept in a secure place for analysis.

The information recorded in the medical records form included the kind of substances (pharmaceutical/non-pharmaceutical), gender, age groups, type of exposure (intentional, unintentional), route of exposure (inhalation, ingestion, skin, intravenous, combination of two routes), education, history of underlying somatic diseases (diabetes, cardiac, renal, pulmonary, others), addiction (opioids, stimulants, alcohol, cigarettes, and others), previous history of attempted suicide, suicide in the family, previous self-harming, clinical manifestations on admission, treatment outcome (recovery or death), time from exposure to the hospital visit, and average duration of hospitalization.

### Definitions

The kind of substances was categorized into two groups: (1) pharmaceuticals [central nervous system drugs (antidepressants, antipsychotics, sedative-hypnotics), analgesics, cardiovascular drugs, and other medications], and (2) non-pharmaceuticals [illegal opioids, stimulants, alcohols, others (organic solvents, halogen derivatives corrosive chemicals, detergents, metals, organic chemicals, carbon monoxide, pesticides, mushroom poisoning, envenomation)].

Clinical examinations included the level of consciousness, skin examination, pupil size, and abdominal, cardiovascular, and respiratory system examination. Nausea, vomiting, diarrhea, and abdominal pain were considered manifestations of abnormal gastrointestinal involvement. Palpitation, chest pain, and hemodynamic changes (tachycardia, bradycardia, hypotension, hypertension, and arrhythmia) were defined as abnormal cardiovascular manifestations. Dyspnea, cough, increased bronchi secretion, and abnormal lung auscultation were considered abnormal respiratory functions. Type of exposure was defined as intentional and unintentional based on the history obtained from the patient or relatives, as well as psychiatric evaluation. Patients were also divided into two groups based on age (< 20 years, and ≥ 20 years).

### Statistical analysis

Data analyses were performed using the Statistical Package for the Social Sciences (SPSS) software for Windows (SPSS Inc., Chicago, IL, USA) version 22. The normality of the data was checked using the Kolmogorov–Smirnov test. For continuous variables, mean and standard deviation (SD) for normally distributed data and median with IQR (Inter quartile rang (first-third quartile) for non- normally distributed data were reported. Frequency and percentages were reported for categorical variables. Chi-square or Fisher exact tests were used for comparing categorical variables between groups and independent samples T-test were used for comparing normally distributed and Mann–Whitney for non-normal distributed data. All aforementioned analyses were performed in lower than 20 and more than 20 years subgroups. The distribution of poisoning rate was reported separately in age and sex groups and compared by using chi-square/Fisher exact test. For evaluating the predictors of mortality among studied participants we first assessed the association each potential predictor with mortality in univariate analysis by chi-squared or independent samples *t*-test/Mann–Whitney *U* test and those with p-value < 0.05 entered in multivariable logistic regression analysis. Simple and multivariable (backward stepwise) binary logistic regression was used for quantifying the association of predictors of mortality by odds ratio (OR) and 95% confidence interval (95% CI) for OR. Predictors with P < 0.05 in univariate analyses were entered in multivariable logistic regression and its viability was checked by using classification table (correct classification rate and Hosmer–Lemeshow goodness of fit test). A P-value less than 0.05 was considered statistically significant.

### Ethics approval and consent to participate

This research has been performed in accordance with the Declaration of Helsinki and has been approved by the ethics committee of Isfahan University of Medical Sciences (Ethics code: IR.MUI.MED.REC.1397.316).

## Results

**Figure 1:**
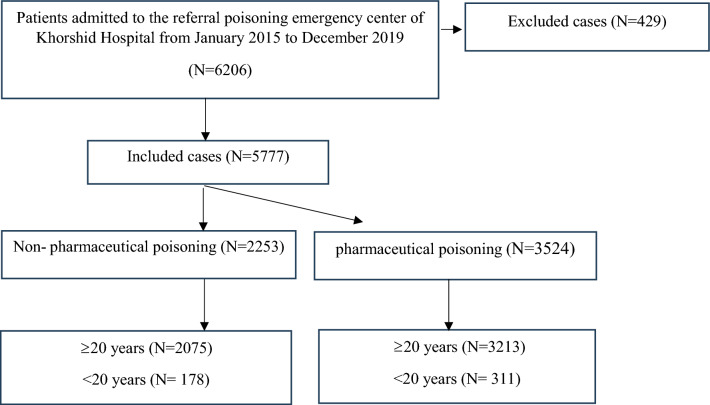
Distribution of patients in the study groups.

During the study period, a total of 6206 patients with acute poisoning were admitted to the referral poisoning emergency center of Khorshid Hospital. Of these, 429 were excluded, leaving 5777 patients who were evaluated. Of the evaluated patients, 3524 (61%) cases were found to be intoxicated by pharmaceuticals, including multidrug toxicity (n = 2453), central nervous system medications (n = 661), analgesic (n = 204), and other medications (n = 206); while 2253 cases (39%) were non-pharmaceutical poisoning including illegal opioids (n = 1243), stimulants (n = 94), alcohols (n = 134), pesticides (n = 467), and others (n = 315) (Fig. 1). such as organic solvents, halogen derivatives, corrosive chemicals, detergents, metals, organic chemicals, carbon monoxide, mushroom poisoning, and envenomation.

Demographic and toxicological data based on pharmaceutical/non-pharmaceutical poisoning are presented in Table 1 and Supplementary Table 1. The type of exposure was intentional in 82.9% and 46.2% of the pharmaceutical and non-pharmaceutical poisoning groups, respectively (P < 0.001). Patients with non-pharmaceutical poisoning were mostly men and had a higher age compared to those with pharmaceutical poisoning, who were mostly women and younger. The place of poisoning, marriage, history of addiction, previous suicide attempts, and history of psychiatric disorders were significantly different between pharmaceutical and non-pharmaceutical poisoning patients (P < 0.001). Patients with pharmaceutical poisoning had more history of previous psychiatric disease, while history of addiction was more observed in patients with non- pharmaceutical poisoning.

**Table 1:** Principal demographic and toxicological data based on pharmaceutical/non-pharmaceutical poisoning in all patients.

Variables		Total (N = 5777)	pharmaceutical poisoning (N = 3524)	Non-pharmaceutical poisoning (N = 2253)	P-value
Gender	Men	2913 (50.4)	1313 (37.3)	1600 (71)	< 0.001
	Women	2864 (49.6)	2211 (62.7)	653 (29)	
Age (year), Mean ± SD (minimum–maximum)		34.30 ± 13.66 (4–99)	32.31 ± 11.69 (4–98)	37.45 ± 15.79 (5–99)	< 0.001
Marriage	Married	3227 (55.9)	1899 (53.9)	1328 (58.9)	< 0.001
	Single	2550 (44.1)	1625 (46.1)	925 (41.1)	
Route of exposure	Oral	5329 (92.1)	3480 (98.8)	1849 (82.1)	< 0.001
	inhalation	114 (2)	5 (0.1)	109 (4.8)	
	injection	33 (0.6)	2 (0.1)	31 (7)	
	Skin	20 (0.3)	0 (0)	20 (0.4)	
	Combination	42 (0.7)	8 (0.2)	8 (0.9)	
	Unknown	239 (4.1)	29 (0.8)	119 (4.8)	
Type of exposure	Intentional	3964 (68.6)	2923 (82.9)	1041 (46.2)	< 0.001
	Unintentional	1813 (31.4)	601 (17.1)	1212 (53.8)	
History of addiction	Yes	1775(30.7)	691 (19.6)	1084 (48.1)	< 0.001
	No	3711 (64.2)	2656 (75.4)	1055 (46.8)	
	No data available	291 (5)	177 (5)	114 (5.1)	
Previous psychiatric disorder	Yes	1147 (19.9)	843 (23.9)	304 (13.5)	< 0.001
	No	4033 (69.8)	2357 (66.9)	1676 (74.4)	
	No data available	597 (10.3)	324 (9.2)	273 (12.1)	

The results are presented as number (percent) per variable or mean ± SD (minimum–maximum) where applicable. Categorical variables were compared between groups with Fisher’s exact or Chi-square tests where appropriate. And continuous variables were compared with independent samples *T*-test. The P value less than 0.05 were considered statistically significant.

The patients were divided into two groups: ≥ 20 years and < 20 years. The results regarding demographic and toxicological data based on these two groups are presented in Supplementary Table 2. In the patients: ≥ 20 years old, frequency of addiction was 21.2% and 52% in pharmaceutical and non-pharmaceutical poisoning, respectively (P < 0.001).

**Figure 2:**
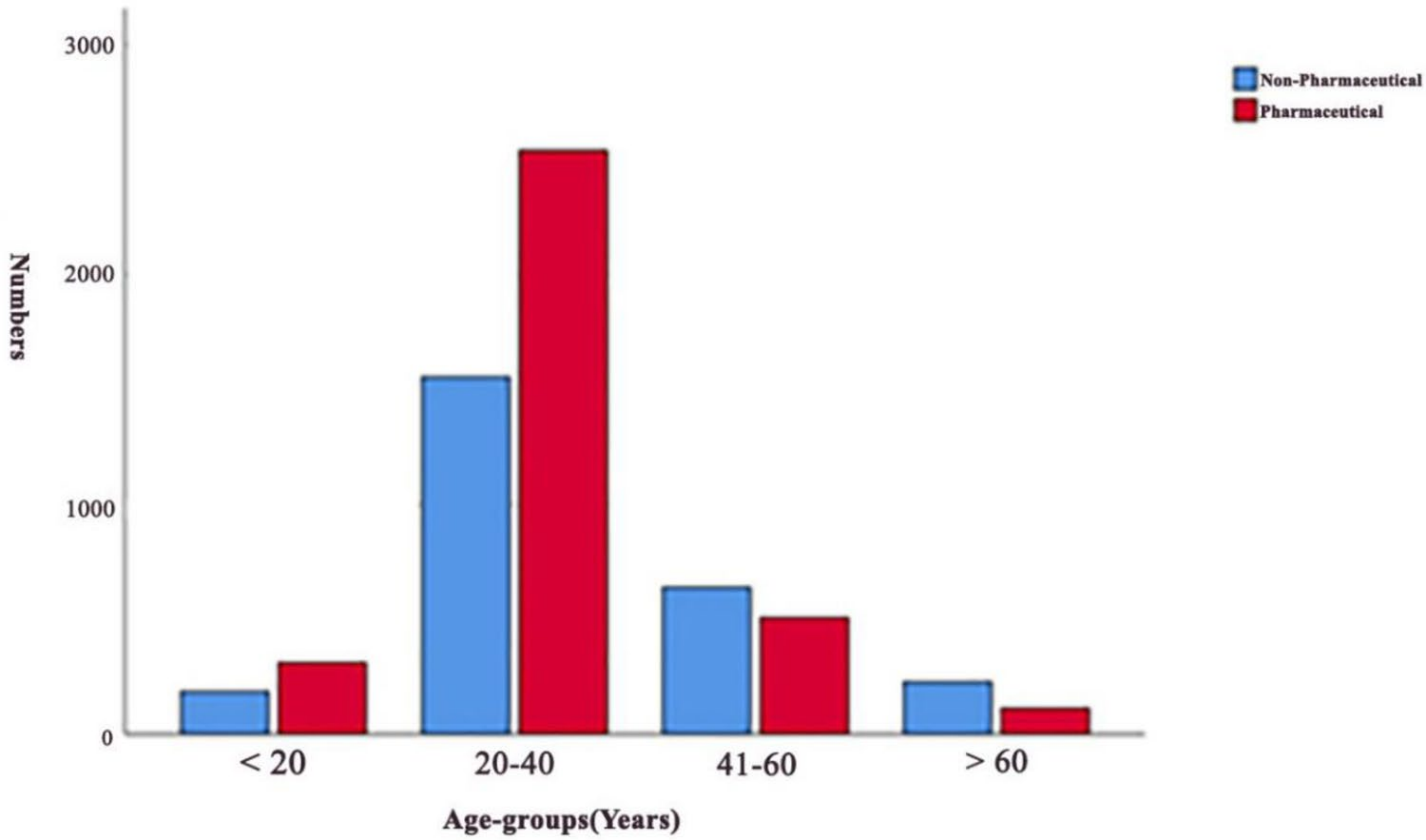
Comparison of poisoning (pharmaceutical/non-pharmaceutical) based on different age groups.

Poisoning was most common in the 20–40 age groups. A comparison of poisoning (pharmaceutical /non-pharmaceutical) based on different age groups is shown in Fig. 2. The majority of patients were between the ages of 20–40. Although non-pharmaceutical poisoning was more common in patients under 40, pharmaceutical poisoning was more observed in patients over 40 (Fig. 2).

Clinical manifestations and outcomes are presented in Table 2. The majority of patients were alert on admission; however, there was a significant difference between the two groups in terms of their level of consciousness (P < 0.001). Abnormal cardiovascular and respiratory system manifestations were more observed in patients with non-pharmaceutical poisoning. There was also a significant difference in the median time intervals from poisoning to receiving the first treatment between the two groups (2 h in pharmaceutical poisoning versus 2.50 h in non-pharmaceutical poisoning (P = 0.001). Additionally, the time interval from poisoning to death was shorter in patients with pharmaceutical poisoning (4 h) compared to patients with non-pharmaceutical poisoning (6.56 h) (P = 0.017). Most patients in both groups survived without complications. However, there were 14 deaths (0.4%) in the pharmaceutical and 48 deaths (2.1%) in the non-pharmaceutical group (P < 0.001).

**Table 2:** Clinical manifestations and outcome based on pharmaceutical and non-pharmaceutical poisoned patients.

Variables		Total (N = 5777)	Pharmaceutical poisoning (N = 3524)	Non-pharmaceutical poisoning (N = 2253)	P-value*
Level of consciousness	Alert	3300 (57.1)	1943 (55.1)	1357 (60.2)	0.001
	Lethargic	1464 (25.3)	1039 (29.5)	425 (18.9)	
	Obtundation	324 (5.6)	169 (4.8)	155 (6.9)	
	Stupor	175 (3)	105 (3)	70 (3.1)	
	Coma	97 (1.7)	52 (1.5)	45 (2)	
	Agitation	139 (2.4)	63 (1.8)	76 (3.4)	
	No data available	278 (4.8)	153 (4.3)	125 (5.5)	
Skin examination	Normal	5004 (86.6)	3143 (89.2)	1861 (82.6)	< 0.001
	Warm and dry	90 (1.6)	48 (1.4)	42 (1.9)	
	Flushing	28 (0.5)	7 (0.2)	21 (0.9)	
	Icterus	18 (0.3)	10 (0.3)	8 (0.4)	
	Cold and wet	177 (3.1)	87 (2.5)	90 (4.0)	
	Sweating	84 (1.5)	28 (0.8)	56 (2.5)	
	No data available	376 (6.5)	201 (5.7)	175 (7.8)	
Pupil size	Normal	3739 (64.7)	2460 (69.8)	1279 (56.8)	< 0.001
	Mydriasis	703 (12.2)	505 (14.3)	198 (8.8)	
	Miosis	964 (16.7)	337 (9.6)	627 (27.8)	
	No data available	371 (6.4)	849 (24.1)	149 (6.6)	
GI manifestations	Abnormal	56 (1)	22(0.6)	34 (1.5)	0.001
	Normal	4280 (74.1)	2653 (75.3)	1627 (72.2)	
	No data available	1441 (24.9)	849 (24.1)	592 (41.1)	
Cardiovascular manifestations	Abnormal	846 (14.6)	482 (13.7)	364 (16.2)	0.007
	Normal	3511 (60.8)	2179 (61.8)	1332 (59.1)	
	No data available	1420 (24.6)	863 (24.5)	557 (24.7)	
Respiratory system manifestations	Abnormal	147 (2.5)	43 (1.2)	104 (4.6)	< 0.001
	Normal	4785 (82.8)	2978 (84.5)	1807 (80.2)	
	No data available	845 (14.6)	503 (14.3)	342 (15.2)	
Endotracheal intubation	Yes	547 (9.5)	346 (9.8)	201 (8.9)	0.256
	No	5230 (90.5)	3178 (90.2)	2025 (91.1)	
The time interval between suspected poisoning and the first treatment (hours); (Median)(IQR)		2.5 (1–7.9)	3 (2–12)	2 (1–6)	0.001
Time interval from admission to death (hours); (Median) (IQR)		7.5 (2.4–18.5)	6 (3–13)	8.5 (2.1–25.5)	0.017
Outcome	Recovery without complication	5685 (58.4)	3496 (99.2)	2189 (97.19)	< 0.001
	Recovery with complication	30 (0.5)	14 (0.4)	16 (0.71)	
	Death	62 (1.1)	14 (0.4)	48 (2.1)	

The results are presented as number (percent) or mean ± SD (or median (IQR:first quartile-third quartile).

*Categorical variables were compared between groups with Fisher’s exact or Chi-square tests or Mann–Whitney *U* test for non-normal continuous data, where appropriate. Time to treatment and time to death were compared using Mann–Whitney. The P value less than 0.05 were considered statistically significant. GI, Gastrointestinal; abnormal gastrointestinal manifestations (nausea, vomiting, diarrhea, and abdominal pain); abnormal cardiovascular manifestations (tachycardia, 2.4bradycardia, hypotension, hypertension, and arrhythmia); abnormal respiratory manifestations (dyspnea, cough, increased bronchi secretion, and abnormal lung auscultation).

Clinical manifestations and outcomes in patients equal or older than 20 years and less than 20 years are presented in Supplementary Table 3. There was a statistically significant difference with respect to the clinical manifestations and outcome in ≥ 20 years’ patients. Gastric lavage and activated charcoal administration were performed in 33% and 69.9% of the patients respectively which was not different with respect to non-pharmaceutical/ pharmaceutical poisoning cases.

The results of binary logistic regression showed that none of the variables were risk factors for mortality in children, however, non-pharmaceutical poisoning was a risk factor for worse outcomes in adults, [OR, 3.14; 95% CI 1.39–7.10, P = 0.006] (Table 3).

**Table 3:** Predictive factors for mortality in adults’ patients.

Model	Variables	OR (95% CI)	P-value
Crude model	Non-pharmaceutical poisoning	5.29 (2.90–9.64)	< 0.001
	Male	2.68 (1.51–4.75)	0.001
	Age	1.04 (1.02–1.64)	< 0.001
	Low level of consciousness	8.34 (3.92–17.37)	< 0.001
	Abnormal cardiovascular manifestation	5.31(2.83–9.95)	< 0.001
	Abnormal respiratory manifestation	13.97 (7.14–27.32)	< 0.001
	Abnormal skin manifestations	3.43 (1.52–7.75)	0.003
	Mydriatic pupils	3.13 (1.57–6.25)	0.001
	Miosis pupils	3.32 (1.79–6.16)	< 0.001
Model 1	Non-pharmaceutical poisoning	3.14 (1.39–7.10)	0.006
	Abnormal cardiovascular manifestation	3.02 (1.23–7.41)	0.016
	Low level of consciousness	7.27 (2.47–21.41)	< 0.001
	Age	1.03 (1.01–1.05)	0.004

Backward stepwise binary logistic regression analysis was employed to calculate the odds ratio (OR) as the estimated predictive factor for mortality; Crude model is from simple binary logistic regression and model 1 represents the results of multivariable binary logistic regression.

*OR* odds ratio, *CI* confidence interval; obtained from simple and backward stepwise multivariable binary logistic regression was used for analysis. Statistical significance at P < 0.05; abnormal gastrointestinal manifestations (nausea, vomiting, diarrhea, and abdominal pain); abnormal cardiovascular manifestations (tachycardia, bradycardia, hypotension, hypertension, and arrhythmia); abnormal respiratory manifestations (dyspnea, cough, increased bronchi secretion, and abnormal lung auscultation).

## Discussion

We conducted a clinical epidemiological investigation in pharmaceutical/non-pharmaceutical poisoning in patients referred to Khorshid Hospital during 2015–2019 in Isfahan, an industrialized province.

In our study, most of the admitted patients were due to pharmaceutical poisoning. In developed countries, the most common cause of acute poisoning is the abuse of commercially available pharmaceuticals^10^. In contrast, in developing countries, insecticides are the most common^11^. In another study in Iran between 2010 and 2018, Mehrpour et al. reported that pharmaceutical medications were the most common poisoning among patients admitted to the intensive care unit^4^. The prevalence of acute poisoning varies in relation to religious, cultural, and geographical contexts and is dynamic given the continued development and varying availability of different xenobiotics^17^.

Our results showed that pharmaceutical poisoning was more common in females which is similar to other studies in Iran^20^. However, non-pharmaceutical poisoning was observed more in men. The social, cultural, or behavioral factors may contribute to these patterns. Increase in psychosocial problems as well as the increase in "acting out" suicide among women^21^, and gender-specific healthcare-seeking behaviors are some related factors^22^. Traditionally, women often assume primary caregiving roles within families and communities, resulting in increased exposure to pharmaceutical substances through medication management responsibilities^22^. Another observed disparity may be attributable to occupational and recreational activities that predispose men to hazardous environments and toxic substances^23,24^. Thus, policymakers should have increased control over the sale of medications without a physician's prescription, organize training workshops to monitor and evaluate drug prevention programs in communities, update regular rules and implemented an effective monitoring system.

In our study, in children, pharmaceutical poisoning was more common in females but non-pharmaceutical poisoning was more observed in males. Kumar and colleagues found that most cases involving household products were in children under 5 years old^25^. Regression analysis revealed that both non-pharmaceutical poisoning and male gender were predictive factors for mortality in all patients. However, non-pharmaceutical poisoning was not found to be a risk factor for mortality in children. This may be because our referral center primarily deals with adult poisoning cases, and some children are admitted to other hospitals for unintentional poisoning.

Similar to other studies^20,26,27^, the highest rate of poisoning was observed among individuals aged 20–40 years. In light of the wide range of risk factors associated with poisoning, it is likely that multiple psychosocial, drug availability and economic factors played a role. Both age groups, 20–40 years and over 40 years, had the highest rates of poisoning. However, the pattern of poisoning differed between the two groups, with pharmaceutical poisoning being more prevalent in the younger age group (20–40 years) and non- pharmaceutical poisoning being more prevalent in the older age group (over 40 years). Another study found that women aged 19–44 had the highest rate of suicide cases; however, there was no significant difference between gender and type of exposure^28,29^. Older patients may use more pesticides for poisoning compared to younger patients who use drugs for suicide purposes^30^. Occupational pesticide exposure may explain the higher use of pesticides by older adults for suicide. This finding is consistent with other studies^31–33^.

The majority of poisoning cases in both pharmaceutical and non-pharmaceutical groups involved married individuals. This finding is consistent with a study conducted in Berhampur, Orissa, which examined the pattern of poisoning cases^34^. Economic pressure, which is a major motivating factor for suicide in men, increases after marriage, especially in countries experiencing economic tensions^35^.

The results showed the type of exposure was more intentional in the patients with pharmaceutical poisoning. This finding aligns with existing research, as readily available pharmaceuticals are frequently misused for suicide attempts^24^. Self-poisoning by drugs is the most common method of suicide in Asia, particularly in countries like China, India, Pakistan, Bangladesh, and Sri Lanka^36–38^. Studies conducted in high-income countries report varying levels of intentional exposure for pharmaceutical poisoning, ranging from approximately 40 to 70%^39^. This suggests potential differences in cultural factors, access to mental healthcare services, and the availability and types of pharmaceuticals between the different societies. Conversely, route of exposure in non-pharmaceutical poisoning was more unintentional similar to other studies^23^. Although we did not evaluate the specific types of non-pharmaceutical poisoning in our study, accidental poisoning with household products may contribute to a higher incidence of unintentional poisoning in the non-drug group^40^.

The history of addiction was higher in non-pharmaceutical poisoning compared to pharmaceutical poisoning. In another study, the frequency of addiction differed between the two groups, with patients who had a history of addiction and psychiatric disorders being more likely to experience non-pharmaceutical poisoning^20^. Dragisic et al. reported that psychiatric history and addiction were common among patients who committed suicide^41^. Recently, a systematic review revealed a connection between mental disorders and overdose. The evidence suggests that individuals with mental disorders are more likely to experience both fatal and non-fatal opioid overdoses. However, the causal direction of this relationship remains unclear^42^.

Patients with non-pharmaceutical poisoning exhibited more abnormal manifestations in the gastrointestinal, cardiovascular, and respiratory systems compared to those with pharmaceutical poisoning. However, there was no difference in the history of underlying somatic disease between the two groups. It can be justified as most of the non-pharmaceutical poisoning was due to pesticide poisoning which has detrimental effects on multiple organs. Abnormal cardiovascular and central nervous system manifestations were also predictive factors for mortality. While drugs like beta-blockers, calcium channel blockers, and digoxin are associated with a significant risk of cardiovascular poisoning, non-pharmaceutical poisoning with substances like pesticides and household products can also lead to cardiovascular toxicity^31,32^. Additionally, some household products and pesticides, such as organophosphates, can have toxic effects on the respiratory system, which is the primary route of absorption for inhaled gases and atmospheric particles. The severity of lung poisoning depends on the dose of the toxic substance delivered to the lung tissues, as well as the acute and chronic effects of the toxin. Certain toxins, such as the paraquat herbicide, can cause tissue damage, lesions, and respiratory impairment, including inflammation effects like fibrosis^43^.

Regression analysis also revealed that poisoning with non-pharmaceutical toxins was a predictive factor for mortality. This may be because our referral poisoning center deals with a high number of pesticides poisoning cases, including paraquat and phosphides, which are associated with high mortality rates^32,43^. Pesticide poisoning accounted for 23.39% of non-pharmaceutical poisoning cases.

The implications of high rates of intentional exposure on mental health services and suicide prevention programs in Iran need to be carefully considered. These prevention programs aim to impact everyone, reduce the risk of suicide by eliminating barriers to care, increase awareness on how to assist individuals contemplating suicide, enhance access to resources, and bolster protective factors like social support and coping mechanisms. Public interventions may involve initiatives such as public education campaigns, suicide awareness programs in schools, restrictions on access to means, media training on reporting suicide incidents, and the implementation of crisis response plans and teams in schools. Also, the significant prevalence of pesticide poisoning in Iran offers crucial data for targeted interventions and policy development.

This manuscript suggests areas for future research studies to investigate the underlying causes of observed gender differences in intoxication patterns. Patterns and risk factors of poisoning vary from region to region and over time within the same region. Regular updating of epidemiologic data is necessary to identify trends for specific factors and risk factors, enabling public global health practitioners to develop preventive strategies and assist physicians in treating patients. Our study has provided an example of how to collect such data.

## Conclusions

In conclusion, pharmaceutical poisoning was more common in women, and intentional exposure; whereas non-pharmaceutical poisoning was more observed in men and unintentional exposure. In adults, abnormal clinical manifestations of the gastrointestinal, cardiovascular, and respiratory systems, as well as the higher time interval from suspected poisoning to the first treatment, were observed more in patients with non-pharmaceutical poisoning, while the history of the underlying somatic disease was not different between the two groups. Patients with non-pharmaceutical poisoning had a higher risk of mortality. Knowing predicting factors for mortality may help physicians select patients for ICU care. Although non-pharmaceutical poisoning is accompanied by more clinical symptoms, due to a lack of information related to severity, we cannot draw a definitive conclusion. Many of the patients had been poisoned by pesticides, which are common in our referral center and have a high mortality rate. Given the importance pesticide intoxication, especially in developing countries, meticulous and robust preventive strategies are suggested to be applied. This pattern shows that the incidence of poisonings could easily be reduced through adequate family education and raising social awareness, as well as maintaining healthy life skills and providing psychological support to vulnerable individuals.

### Limitations of the study

The study's retrospective design and single-center nature could limit the generalizability of the results. There was a not-recorded data that we included in supplementary files. Additionally, the study was cross-sectional and both adults and children were evaluated, which may have resulted in different severity of symptoms and outcomes with respect to pharmaceutical and non-pharmaceutical poisoning. Data quality was another important issue, and since the nature of the study was retrospective, we were unable to overcome this limitation. Also given the very low occurrence of the outcome of mortality amongst patients less than 20 years old, the inference that no variables were risk factors for children may not be correct. Rather, it is indeterminable. Therefore, this study did not have enough data on patients less than 20 years old and that more data are needed. Furthermore, we did not evaluate the impact of socioeconomic and psychological factors that are important in understanding this type of poisoning. Moreover, we did not analyze specific types of pharmaceuticals and non-pharmaceuticals agents based on different studied variables. Finally, if our missing data for some variables (that was reported as not-available data) was actually available, or declared by participants we could reach more concise results. Although, our findings are based on data from a referral center, however it is important to note that this may not fully reflect the overall conditions of poisoned individuals in our society. Our data is naturally subject to selection bias, and information bias because of nature of cross-sectional study, although we tried to overcome some of these biases by choosing an appropriate and well-designed protocols for data collection and handling. We also tried to interview with the patients’ family to minimize the selection bias, however not all of them responded. We did not include the specific therapeutic modalities have been used in all patients according the studied groups, which may be a confounding factor.

### Supplementary Information

Supplementary Tables.

## Data Availability

The datasets generated and/or analyzed during the current study are not publicly available due to the nature of patients who attempted suicide but are available from the corresponding author on reasonable request.

## References

[1] Boedeker W, Watts M, Clausing P, Marquez E (2020). The global distribution of acute unintentional pesticide poisoning: Estimations based on a systematic review. BMC Public Health.

[2] The Global burden of disease report 2014, WHO website, International Programme on Chemical Safety: Poisoning Prevention and Management. www.who.int/ipcs/poisons/en (Accessed 9th January 2017).

[3] Mehrpour O, Zamani N, Brent J, Abdollahi M (2013). A tale of two systems: Poisoning management in Iran and the United States. DARU J. Pharm. Sci..

[4] Mehrpour O, Akbari A, Jahani F, Amirabadizadeh A, Allahyari E, Mansouri B (2018). Epidemiological and clinical profiles of acute poisoning in patients admitted to the intensive care unit in eastern Iran (2010 to 2017). BMC Emerg. Med..

[5] Mehrpour O, Abdollahi M (2012). Poison Treatment Centers in Iran.

[6] Hayatbakhsh MM, Oghabian Z, Conlon E, Nakhaee S, Amirabadizadeh AR, Zahedi MJ (2017). Lead poisoning among opium users in Iran: An emerging health hazard. Subst. Abuse Treat. Prev. Policy.

[7] Chelkeba L, Mulatu A, Feyissa D, Bekele F, Tesfaye BT (2018). Patterns and epidemiology of acute poisoning in Ethiopia: Systematic review of observational studies. Arch. Public Health.

[8] Mehrpour O, Hoyte C, Amirabadizadeh A, Brent J, Consortium TI (2020). Clinical characteristics and time trends of hospitalized methadone exposures in the United States based on the Toxicology Investigators Consortium (ToxIC) case registry: 2010–2017. BMC Pharmacol. Toxicol..

[9] Abd-Elhaleem ZAE, Al MB (2014). Pattern of acute poisoning in Al Majmaah region, Saudi Arabia. Am J Clin Exp Med..

[10] Shadnia S, Esmaily H, Sasanian G, Pajoumand A, Hassanian-Moghaddam H, Abdollahi M (2007). Pattern of acute poisoning in Tehran-Iran in 2003. Hum. Exp. Toxicol..

[11] Abdollahi M, Jalali N, Sabzevari O, Hoseini R, Ghanea T (1997). A restrospective study of poisoning in Tehran. J. Toxicol. Clin. Toxicol..

[12] Sarkar S, Goswami B, Sengupta B, Sengupta S, Bhattacharjee B, Harish J (2022). Epidemiological profile of acute poisoning cases and its outcome in a North Eastern teaching hospital of India. Toxicology.

[13] Hadeiy SK, Parhizgar P, Hassanian-Moghaddam H, Zamani N, Khoshkar A, Kolahi A-A (2022). Trends of acute drug and chemical toxicities in adults and adolescents in Tehran, Iran between 2012 and 2018: A retrospective chart review. Drug Chem. Toxicol..

[14] Alinejad S, Zamani N, Abdollahi M, Mehrpour O (2017). A narrative review of acute adult poisoning in Iran. Iran. J. Med. Sci..

[15] Mehrpour O, Farzaneh E, Abdollahi M (2011). Successful treatment of aluminum phosphide poisoning with digoxin: A case report and review of literature. Int. J. Pharmacol..

[16] Deng J-F. The challenge of poison control centers in Asia-Pacific. 勞工安全衛生研究季刊. 18(2), 231–42 (2010).

[17] Al-Jahdali H, Al-Johani A, Al-Hakawi A, Arabi Y, Ahmed QA, Altowirky J (2004). Pattern and risk factors for intentional drug overdose in Saudi Arabia. Can. J. Psychiatry.

[18] Goel A, Aggarwal P (2007). Pesticide poisoning. Natl. Med. J. India.

[19] Organization WH. International Statistical Classification of Diseases and related health problems: Alphabetical index: World Health Organization (2004).

[20] Naseri K, Kiani Z, Sajadi ZS, Mehrpour O, Javadmoosavi SY, Forouzanfar F (2023). Pharmaceutical toxicity is a common pattern of inpatient acute poisonings in Birjand City, East of Iran. Sci. Rep..

[21] Mohammad Hosseini S, Karimi Z, Afrasiyabifar A, Naeimi E, Moghimi M, Sadat S (2012). Causes of acute poisoning hospital admission in shahid beheshti hospital of yasuj, 2008. Armaghane Danesh.

[22] Miller MA (2001). Gender-based differences in the toxicity of pharmaceuticals—The Food and Drug Administration's perspective. Int. J. Toxicol..

[23] Biswas A, Harbin S, Irvin E, Johnston H, Begum M, Tiong M (2021). Sex and gender differences in occupational hazard exposures: A scoping review of the recent literature. Curr. Environ. Health Rep..

[24] Azizpour Y, Asadollahi K, Sayehmiri K, Kaikhavani S, Abangah G (2016). Epidemiological survey of intentional poisoning suicide during 1993–2013 in Ilam Province, Iran. BMC Public Health.

[25] Bharath Kumar C, Chowdhury SD, Ghatak SK, Sreekar D, Kurien RT, David D (2019). Immediate and long-term outcome of corrosive ingestion. Indian J. Gastroenterol..

[26] Eazari E, Nazari S, Ebnehosseini Z, Akhavan R, Tabesh H (2021). Analysis of the pattern of poisoning in patients admitted to a large teaching hospital in Iran. J. Biostat. Epidemiol..

[27] Banaye Yazdipour A, Moshiri M, Dadpour B, Sarbaz M, Heydarian Miri H, Hajebi Khaniki S (2022). The trend of top five types of poisonings in hospitalized patients based on ICD-10 in the northeast of Iran during 2012–2018: A cross-sectional study. Health Sci. Rep..

[28] Masoumi G, Ganjei Z, Teymoori E, Sabzghabaee AM, Yaraghi A, Akabri M (2013). Evaluating the prevalence of intentional and unintentional poisoning in vulnerable patients admitted to a referral hospital. J. Isfahan Med. Sch..

[29] Mirmoghtadaei P, Eizadi-Mood N, Mahvari R, Mohammadbeigi E, Savari MA, Feizi A (2022). Intentional versus accidental pesticide poisoning in hospitalized patients in poisoning referral center in Isfahan, Iran: A cross sectional study. Int. J. Med. Toxicol. Legal Med..

[30] Song SJ, Park GJ, Lee JH, Kim SC, Kim H, Lee SW (2020). The characteristics of elderly individuals who attempted suicide by poisoning: A nationwide cross-sectional study in Korea. J. Korean Med. Sci..

[31] Eizadi-Mood N, Lalehzar SS, Niknam S, Mahvari R, Mirmoghtadaee P, Meamar R (2022). Toxico-clinical study of patients poisoned with household products; a two-year cross-sectional study. BMC Pharmacol. Toxicol..

[32] Dorooshi G, Mirzae M, Fard NT, Zoofaghari S, Mood NE (2021). Investigating the outcomes of aluminum phosphide poisoning in khorshid referral hospital, Isfahan, Iran: A retrospective study. J. Res. Pharm. Pract..

[33] Rostami M, Jalilian A, Ghadirzadeh MR, Nazparvar B, Rezaei-Zangeneh R, Karamouzian M (2023). Bayesian spatial analysis of age differences and geographical variations in illicit-drug-related mortality in the Islamic Republic of Iran. East. Mediterr. Health J..

[34] Singh VP, Sharma B, Harish D, Vij K, editors. A ten year study of poisoning cases in a tertiary care hospital. Indian Congress of Forensic Medicine & Toxicology (2004).

[35] Nair PK, Revi NG (2015). One-year study on pattern of acute pharmaceutical and chemical poisoning cases admitted to a tertiary care hospital in Thrissur, India. Asia Pac. J. Med. Toxicol..

[36] Lee J-W, Hwang I-W, Kim J-W, Moon H-J, Kim K-H, Park S (2015). Common pesticides used in suicide attempts following the 2012 paraquat ban in Korea. J. Korean Med. Sci..

[37] Konradsen F, van der Hoek W, Peiris P. Reaching for the bottle of pesticide—a cry for help. Self-inflicted poisonings in Sri Lanka. Social science & medicine. 2006;62(7):1710–9. 10.1016/j.socscimed.2005.08.020 16165259

[38] Najafi F, Beiki O, Ahmadijouybari T, Amini S, Moradinazar M, Hatemi M (2014). An assessment of suicide attempts by self-poisoning in the west of Iran. J. Forensic Leg. Med..

[39] Cowans C, Love A, Tangiisuran B, Jacob SA (2023). Uncovering the hidden burden of pharmaceutical poisoning in high-income and low-middle-income countries: A scoping review. Pharmacy.

[40] Eizadi-Mood N, Lalehzar SS, Niknam S, Mahvari R, Mirmoghtadaee P, Meamar R (2022). Toxico-clinical study of patients poisoned with household products; a two-year cross-sectional study. BMC Pharmacol. Toxicol..

[41] Dragisic T, Dickov A, Dickov V, Mijatovic V (2015). Drug addiction as risk for suicide attempts. Mater. Socio-med..

[42] van Draanen J, Tsang C, Mitra S, Phuong V, Murakami A, Karamouzian M (2022). Mental disorder and opioid overdose: A systematic review. Soc. Psychiatry Psychiatr. Epidemiol..

[43] Isfahani SN, Farajzadegan Z, Sabzghabaee AM, Rahimi A, Samasamshariat S, Eizadi-Mood N (2019). Does hemoperfusion in combination with other treatments reduce the mortality of patients with paraquat poisoning more than hemoperfusion alone: A systematic review with meta-analysis. J. Res. Med. Sci. Off. J. Isfahan Univ. Med. Sci..

