# Supplementary material for: Clinico-epidemiological evaluation of pharmaceutical/non-pharmaceutical poisoning in a referral poisoning emergency in the Central part of Iran
Source: Sci Rep. 2024 May 7;14:10493. doi: 10.1038/s41598-024-61411-w (PMC11076496; doi:10.1038/s41598-024-61411-w)
Supplement: Supplementary file 1 — Supplementary Tables. [file 41598_2024_61411_MOESM1_ESM.docx]

**Supplementary Table 1:** Demographic and toxicological data based on drug/non drug poisoning

| Variables | | Total  (N=5777) | Drug poisoning  (N=3524 ) | Non-drug poisoning  (N= 2253 ) | P-value |
| --- | --- | --- | --- | --- | --- |
| Number of children | 0 | 2992(51.8) | 1951(55.4) | 1041(46.2) | <0.001 |
|  | 1-4 | 1203(20.8) | 683(19.4) | 520(23.1) |  |
|  | 5-8 | 95(1.6) | 33(0.9) | 62(2.8) |  |
|  | 9-12 | 7(0.1) | 1(0) | 6(0.3) |  |
|  | No data available | 1480(25.6) | 856(24.3) | 624(42.2) |  |
| Education | No formal education | 31(0.5) | 20(0.6) | 11(0.5) | 0.792 |
|  | High school | 111(1.9) | 70(2) | 41(1.8) |  |
|  | Diploma | 160(2.8) | 105(3) | 55(2.4) |  |
|  | University | 67(1.1) | 42(1.2) | 25(1.1) |  |
|  | No data available | 5408(93.6) | 3287(93.3) | 2121(94.1) |  |
| Place of poisoning | Home | 3699(64.0) | 2508(71.2) | 1191(52.9) | <0.001 |
|  | At Work | 210(3.6) | 63(1.8) | 147(6.5) |  |
|  | Other places | 352(6.1) | 146(4.1) | 206(9.1) |  |
|  | No data available | 1516(26.2) | 807(22.9) | 709(31.5) |  |
| Kind of Addiction | Opium | 312(5.4) | 77(2.2) | 235(10.4) | <0.001 |
|  | Heroin | 94(1.6) | 34(1) | 60(2.7) |  |
|  | Cigarettes | 320(5.5) | 213(6) | 107(4.7) |  |
|  | Alcohol | 54(0.9) | 23(0.7) | 31(1.4) |  |
|  | Others | 280(4.8) | 117(3.3) | 163(7.2) |  |
|  | Combination | 574(9.9) | 189(5.4) | 385(39.2) |  |
|  | No data available | 4143(71.7) | 2871(81.5) | 1272(56.5) |  |
| Previous attempted of suicide | Yes | 941(16.3) | 710(20.1) | 231(10.3) | <0.001 |
|  | No | 3483(59.5) | 2048(58.1) | 1390(61.7) |  |
|  | No data available | 1398(24.2) | 766(21.7) | 632(28.1) |  |
| Criminal record | Yes | 217(3.8) | 73(2.1) | 144(6.4) | <0.001 |
|  | No | 4449(77.0) | 2800(79.5) | 1649(73.2) |  |
|  | No data available | 1111(19.2) | 651(18.5) | 460(20.4) |  |
| Previous self-harming | Yes | 353(6.1) | 247(17.0) | 106(4.7) | <0.001 |
|  | No | 3011(52.1) | 1858(52.7) | 1153(51.2) |  |
|  | No data available | 2413(41.8) | 1419(40.3) | 994(44.1) |  |
| History of suicide in family | Yes | 246(4.3) | 192(5.4) | 54(2.4) | <0.001 |
|  | No | 4403(76.2) | 2710(76.9) | 1693(51.5) |  |
|  | No data available | 1128(19.5) | 622(17.7) | 506(22.5) |  |
| History of underlying somatic disease | Yes | 1473(25.5) | 930(26.4) | 543(4.1) | 0.096 |
|  | No | 2955(51.2) | 1795(50.9) | 1160(74.4) |  |
|  | No data available | 1349(23.4) | 799(22.7) | 550(24.4) |  |

The results are presented as number (percent); Categorical variables were compared between groups with Fisher’s exact or Chi-square tests where appropriate. The P value less than 0.05 were considered statistically significant.

**Supplementary Table 2:** Demographic and toxicological data in adult and children based on drug/non drug poisoning

| Variables | | ≥ 20 years  (N=5288) | | | | < 20 years  (N= 489) | | | |
| --- | --- | --- | --- | --- | --- | --- | --- | --- | --- |
|  |  | Total | Drug (N=3213) | Non-drug  (N=2075) | P-value | total | Drug  (N=311) | Non-drug  (N= 178) | P-value |
| Gender | female | 2567(48.5) | 1991(62.0) | 576(27.8) | <0.001 | 297(60.7) | 220(70.7) | 77(43.3) | <0.001 |
|  | male | 2721(51.5) | 1222(38.0) | 1499(72.2) |  | 192(39.3) | 91(29.3) | 101(56.7) |  |
| Route of exposure | Oral | 4857(93.6) | 3172(99.4) | 1685(84.7) | <0.001 | 472(97.7) | 308(99.4) | 164(94.8) | 0.001 |
|  | inhalation | 112(2.2) | 4(0.1) | 108(5.4) |  | 2(0.4) | 1(0.6) | 1(0.3) |  |
|  | injection | 33(0.6) | 31(1.5) | 2(0.1) |  |  |  |  |  |
|  | Skin | 18(0.3) | 0(0.00) | 18(0.9) |  | 2(0.4) | 0(0.00) | 2(1.2) |  |
|  | Combination | 41(0.8) | 33(1.7) | 8(0.3) |  | 1(0.2) | 0(0.00) | 1(0.6) |  |
|  | Unknown | 118(2.3) | 4(0.1) | 114(5.7) |  | 6(1.2) | 1(0.3) | 5(2.9) |  |
| Type of exposure | Intentional | 3700(70.0) | 2705(84.2) | 995(48.0) | <0.001 | 264(54.0) | 218(70.1) | 46(25.8) | < 0.001 |
|  | Unintentional | 1588(30.0) | 508(15.8) | 1080(52) |  | 225(46.0) | 93(29.9) | 132(74.2) |  |
| History of Addiction | Yes | 1761(33.3) | 682(21.2) | 1079(52.0) | <0.001 | 14(2.9) | 9(2.9) | 5(2.8) | 0.390 |
|  | No | 3258(61.6) | 2365(74.6) | 893(43.0) |  | 453(92.6) | 291(93.6) | 162(91.0) |  |
|  | No Data available | 269(5.1) | 166(5.2) | 103(5.0) |  | 22(4.5) | 11(3.5) | 11(6.2) |  |
| Previous Psychiatric  disorder | Yes | 1099(20.8) | 801(24.9) | 298(14.4) | <0.001 | 48(9.8) | 42(13.5) | 6(3.4) | <0.001 |
|  | No | 3627(67.6) | 2106(65.5) | 1521(73.3) |  | 406(83.0) | 251(80.7) | 155(87.1) |  |
|  | No Data available | 562(10.6) | 306(9.5) | 256(12.3) |  | 35(7.2) | 18(5.8) | 17(9.6) |  |
| Number of children | 0 | 2543(48.1) | 1663(51.8) | 880(42.4) | <0.001 | 449(91.8) | 288(92.6) | 161(90.4) | 0.190 |
|  | 1-4 | 1201(22.7) | 683(21.3) | 518(25.0) |  | 2(0.4) | 0(0) | 2(1.1) |  |
|  | 5-8 | 95(1.8) | 33(1.0) | 62(3.0) |  | - | - | - |  |
|  | 9-12 | 7(0.1) | 6(0.3) | 1(0.0) |  | - | - | - |  |
|  | No Data available | 1442(27.3) | 833(25.9) | 609(29.3) |  | 38(7.8) | 23(7.4) | 15(8.4) |  |
| Marriage | Married | 3166(59.9) | 1856(57.8) | 1309(63.1) | <0.001 | 62(12.7) | 43(13.8) | 19(10.7) | 0.397 |
|  | Single | 2123(40.1) | 1357(42.2) | 766(26.9) |  | 427(87.3) | 268(86.2) | 159(89.3) |  |
| Education | No formal education | 24(0.5) | 16(0.5) | 8(0.4) | 0.857 | 7(1.4) | 4(1.3) | 3(1.7) | 0.432 |
|  | High school | 98(1.9) | 62(1.9) | 36(1.7) |  | 13(2.7) | 8(2.6) | 5(2.8) |  |
|  | Diploma | 151(2.9) | 97(3.0) | 54(2.6) |  | 9(1.8) | 8(2.6) | 1(0.6) |  |
|  | University | 67(1.3) | 42(1.3) | 25(1.2) |  |  |  |  |  |
|  | No Data available | 4948(93.6) | 2996(93.2) | 1952(94.1) |  | 460(94.1) | 291(93.6) | 169(94.9) |  |
| Place of poisoning | Home | 3343(63.2) | 2275(70.8) | 1068(51.5) | <0.001 | 356(72.8) | 233(74.9) | 123(69.1) | < 0.001 |
|  | At Work | 206(3.9) | 61(1.9) | 145(7) |  | 4(0.8) | 2(0.6) | 2(1.1) |  |
|  | Other places | 329(6.2) | 131(4.1) | 198(9.5) |  | 23(4.7) | 15(4.8) | 8(4.5) |  |
|  | No Data available | 1410(26.7) | 746(23.2) | 664(32.0) |  | 106(21.7) | 61(19.6) | 45(25.3) |  |
| Previous attempted of suicide | Yes | 898(17.0) | 676(21.0) | 222(10.7) | <0.001 | 43(8.8) | 34(10.9) | 9(5.1) | 0.042 |
|  | No | 3128(59.2) | 1850(57.6) | 1278(61.6) |  | 310(63.4) | 198(63.7) | 112(62.9) |  |
|  | No Data available | 1262(23.9) | 687(21.4) | 575(27.7) |  | 136(27.8) | 79(25.4) | 57(32.0) |  |
| Criminal record | Yes | 215(4.1) | 73(2.3) | 142(6.8) | <0.001 | 2(0.4) | 0(0.0  0 | 2(1.1) | 0.245 |
|  | No | 4028(76.2) | 2531(88.8) | 1497(72.1) |  | 421(86.1) | 269(86.5) | 152(85.4) |  |
|  | No Data available | 1045(19.8) | 609(19.0) | 436(21.0) |  | 66(13.5) | 42(13.5) | 24(13.5) |  |
| Previous self-harming | Yes | 331(6.3) | 227(7.1) | 104(5.0) | 0.001 | 22(4.5) | 20(6.4) | 2(1.1) | 0.014 |
|  | No | 2735(51.7) | 1683(52.4) | 1052(50.7) |  | 276(56.4) | 175(56.3) | 101(56.7) |  |
|  | No Data available | 2222(42.0) | 1303(40.6) | 919(44.3) |  | 191(39.1) | 116(37.3) | 75(42.1) |  |
| History of suicide in family | Yes | 222(4.2) | 170(5.3) | 52(2.5) | <0.001 | 24(4.9) | 22(7.1) | 2(1.1) | 0.004 |
|  | No | 4030(76.2) | 2474(77.0) | 1556(75.0) |  | 373(76.3) | 236(75.9) | 137(77.0) |  |
|  | No Data available | 1036(19.6) | 569(17.7) | 467(22.5) |  | 92(18.8) | 53(17.0) | 39(21.9) |  |
| History of underlying somatic disease | Yes | 1405(26.6) | 877(27.3) | 528(37.6) | 0.176 | 68(13.9) | 53(17.0) | 15(8.4) | 0.023 |
|  | No | 2643(50.0) | 1606(50.0) | 1037(50.0) |  | 312(63.8) | 189(60.8) | 123(69.1) |  |
|  | No Data available | 1240(23.4) | 730(22.7) | 510(24.6) |  | 109(22.3) | 69(22.2) | 40(22.5) |  |

The results are presented as number (percent); Categorical variables were compared between groups with Fisher’s exact or Chi-square tests where appropriate. The P value less than 0.05 were considered statistically significant.

**Supplementary Table 3:** Clinical manifestations and outcome in adult and children based on drug/non drug poisoning

| variables |  | Adult  (N=5288) | | | | Children  (N= 489) | | | |
| --- | --- | --- | --- | --- | --- | --- | --- | --- | --- |
|  |  | Total | Drug  (N= 3213) | Non-drug  (N= 2075) | P value | Total | Drug  (N=311 ) | Non-Drug  (N=178 ) | P-value |
| Level of consciousness | Alert | 2982(56.4) | 1738(54.1) | 1244(60.0) | <0.001 | 318(65.0) | 205(65.9) | 113(63.5) | 0.161 |
|  | Lethargic | 1356(25.6) | 966(30.1) | 390(18.8) |  | 108(22.1) | 73(23.5) | 35(19.7) |  |
|  | Obtundation | 303(5.7) | 158(4.9) | 145(7) |  | 21(4.3) | 11(3.5) | 10(5.6) |  |
|  | Stupor | 166(3.1) | 98(3.1) | 68(3.3) |  | 9(1.8) | 7(2.3) | 2(1.1) |  |
|  | Coma | 93(1.8) | 49(1.5) | 44(2.1) |  | 4(0.8) | 3(1.0) | 1(0.6) |  |
|  | Agitation | 132(2.5) | 60(1.9) | 72(3.5) |  | 7(1.4) | 3(1.0) | 4(2.4) |  |
|  | Not data available | 256(4,8) | 144(4.5) | 112(5.4) |  | 22(4.5) | 9(2.9) | 13(7.3) |  |
| Skin examination | Normal | 4576(92.5) | 2863(94.5) | 1713(89.4) | <0.001 | 428(94.3) | 280(95.6) | 148(9.9) | 0.05 |
|  | Warm and dry | 78(1.6) | 40(1.3) | 38(2.0) |  | 12(2.6) | 8(2.7) | 4(2.5) |  |
|  | Flushing | 26(0.5) | 6(0.2) | 20(1.0) |  | 2(0.4) | 1(0.3) | 1(0.6) |  |
|  | Icterus | 17(0.3) | 10(0.3) | 7(0.4) |  | 1(0.2) | 0(0.0) | 1(0.6) |  |
|  | Cold and wet | 170(3.4) | 83(2.7) | 87(4.5) |  | 7(1.5) | 4(1.4) | 3(1.9) |  |
|  | Sweating | 80(1.6) | 28(0.9) | 52(2.7) |  | 4(0.9) | 0(0.0) | 4(2.5) |  |
| Pupil size | Normal | 3402(64.3) | 2230(69.4) | 1172(56.5) | <0.001 | 337(68.9) | 230(74) | 107(60.1) | <0.001 |
|  | Mydriasis | 650(12.3) | 467(14.5) | 183(8.8) |  | 53(10.8) | 38(12.2) | 15(8-4) |  |
|  | Miosis | 898(17) | 315(9.8) | 583(28.1) |  | 66(13.5) | 22 (7.1) | 44(7.1) |  |
|  | Not data available | 338(6.4) | 201(6.3) | 137(6.6) |  | 33(6.7) | 21(6.8) | 12(6.7) |  |
| GI manifestations | Normal | 3908(73.9) | 2411(75.0) | 1497(72.1) | <0.001 | 372(76.1) | 242(99.2) | 130(73.0) | 0.291 |
|  | Abnormal | 54(1.0) | 20(0.6) | 34(1.6) |  | 2(0.4) | 2(0.6) | 0(0.0) |  |
|  | Not data available | 1326(25.1) | 782(24.3) | 544(26.2) |  | 115(23.5) | 67(21.5) | 48(27.0) |  |
| Cardiovascular manifestations | Normal | 3220(60.9) | 1992(62.0) | 1228(59.2) | 0.001 | 291(59..5) | 187(60.1) | 104(58.4) | 0.143 |
|  | Abnormal | 759(14.4) | 421(13.1) | 338(16.3) |  | 87(17.8) | 61(19.6) | 26(14.6) |  |
|  | Not data available | 1309(24.8) | 800(61.1) | 509(24.5) |  | 111(22.7) | 63(20.3) | 48(27.0) |  |
| Respiratory system manifestations | Normal | 4363(82.5) | 2705(84.2) | 1658(79.9) | <0.001 | 422(86.3) | 273(86.6) | 149(83.7) | 0.434 |
|  | Abnormal | 143(2.7) | 41(1.3) | 102(4.9) |  | 4(0.8) | 2(0.6) | 2(1.1) |  |
|  | Not data available | 782(14.8) | 467(14.5) | 315(15.2) |  | 63(12.9) | 36(11.6) | 27(15.2) |  |
| Endotracheal Intubation | Yes | 517(9.8) | 326(10.1) | 191(9.2) | 0.260 | 30(6.1) | 20(6.4) | 10(5.6) | 0.71 |
|  | No | 4771(90.2) | 2887(89.9) | 1884(90.8 |  | 459(93.9) | 291(93.6) | 168(94.4) |  |
| The time interval between suspected poisoning and the first treatment (hours); (Median) | | 2 | 2 | 2.75 | <0.001 | 2 | 2 | 1.50 | 0.001 |
| Time interval from admission to death (hours); (Median) | | 5 | 4 | 7 | <0.001 | 6 | 6 | 6 | 0.017 |
| Outcome | recovery without complication | 5198(98.3) | 3185(99.1) | 2013(97) | <0.001 | 487(99.6) | 311(100) | 176(98.9) | 0.173 |
|  | recovery with complication | 29(0.5) | 14(0.4) | 15(0.7) |  | 1(0.2) | 0(0.0) | 1(0.6) |  |
|  | Death | 61(1.2) | 14(0.4) | 47(2.3) |  | 1(0.6) | 0(0.0) | 1(0.6) |  |

The results are presented as number (percent) or mean ± SD (median); Categorical variables were compared between groups with Fisher’s exact or Chi-square tests where appropriate. For non-normally distributed continuous data using the non-parametric, Mann-Whitney test was used. The P value less than 0.05 were considered statistically significant. GI, Gastrointestinal; abnormal gastrointestinal manifestations (nausea, vomiting, diarrhea, and abdominal pain); abnormal cardiovascular manifestations (tachycardia, bradycardia, hypotension, hypertension, and arrhythmia); abnormal respiratory manifestations (dyspnea, cough, increased bronchi secretion, and abnormal lung auscultation)
